# Human-like face pareidolia emerges in deep neural networks optimized for face and object recognition

**DOI:** 10.1371/journal.pcbi.1012751

**Published:** 2025-01-27

**Authors:** Pranjul Gupta, Katharina Dobs

**Affiliations:** 1 Department of Experimental Psychology, Justus Liebig University Giessen, Giessen, Germany; 2 Center for Mind, Brain, and Behavior, Universities of Marburg, Giessen and Darmstadt, Marburg, Germany; National Institute of Mental Health and Neuro Sciences, UNITEDSTATES OF AMERICA

## Abstract

The human visual system possesses a remarkable ability to detect and process faces across diverse contexts, including the phenomenon of face pareidolia—–seeing faces in inanimate objects. Despite extensive research, it remains unclear why the visual system employs such broadly tuned face detection capabilities. We hypothesized that face pareidolia results from the visual system’s optimization for recognizing both faces and objects. To test this hypothesis, we used task-optimized deep convolutional neural networks (CNNs) and evaluated their alignment with human behavioral signatures and neural responses, measured via magnetoencephalography (MEG), related to pareidolia processing. Specifically, we trained CNNs on tasks involving combinations of face identification, face detection, object categorization, and object detection. Using representational similarity analysis, we found that CNNs that included object categorization in their training tasks represented pareidolia faces, real faces, and matched objects more similarly to neural responses than those that did not. Although these CNNs showed similar overall alignment with neural data, a closer examination of their internal representations revealed that specific training tasks had distinct effects on how pareidolia faces were represented across layers. Finally, interpretability methods revealed that only a CNN trained for both face identification and object categorization relied on face-like features—such as ‘eyes’—to classify pareidolia stimuli as faces, mirroring findings in human perception. Our results suggest that human-like face pareidolia may emerge from the visual system’s optimization for face identification within the context of generalized object categorization.

## Author summary

Have you ever seen a face in an inanimate object, like the foam in your coffee? This phenomenon, known as face pareidolia, demonstrates our brain’s remarkable ability to detect faces even when they’re not there. But why does this happen? Our study explores this question by investigating why our brains employ such broadly tuned face detection capabilities. We compared human behavioral and neural responses to those of artificial neural networks trained on various combinations of face and object tasks, including face detection, face identification, and object categorization. Intriguingly, we found that only a network trained on both face identification (recognizing individual faces) and object categorization aligned with human neural responses and relied on face-like features, such as ‘eyes’, to classify pareidolia stimuli as faces. This suggests that face pareidolia may result from the interplay between fine-grained face identification and generalized object categorization processes in the brain. Our findings shed light on the origins of face pareidolia and highlight the potential of using artificial neural networks to understand the complexities of visual perception.

## Introduction

Faces are special to the visual system. Both humans and non-human primates possess a system of cortical regions dedicated to processing faces [1,2], humans detect faces faster than other visual objects [3,4] and faces rank among the most salient visual stimuli for saccades [5,6]. However, the prioritized processing of faces has its trade-offs, such as the occasional misperception of faces in ordinary objects. This phenomenon, known as face pareidolia, has been the subject of intensive investigation in humans [7–9] and monkeys [10,11] over the past few decades. Recent behavioral and neural evidence suggests a two-stage process of face pareidolia processing: an initial coarse face detection mechanism, during which face-like objects (i.e., pareidolia faces) are represented more similarly to faces than objects, followed by a later face-specific processing stage [12,13]. Despite significant progress in understanding the behavioral and neural signatures of face pareidolia processing, the reason why the brain exhibits such a broadly tuned face detection mechanism remains unclear. Here, we ask whether the phenomenon of face pareidolia emerges from an optimization for the fine-grained recognition of faces among objects in the real world.

Recent advances in deep convolutional neural networks (CNNs), which show high representational similarity to the human visual system [14–16], provide an ideal framework for understanding the type of experience and task optimization that leads to a particular observed phenomenon [17–19]. In particular, if a specific human behavioral or neural phenomenon is proposed to result from optimization for a given task, we expect to observe a similar phenomenon in a deep neural network optimized for that same task. This logic has been previously applied to understand the origins of human face processing (see [20] for a review). For instance, several behavioral signatures of human face perception, such as the face inversion effect, other-race effect, familiarity effect, and view-invariant face representations [21–24], have been replicated in CNNs. Critically, this only holds for networks trained on face identification (recognizing individual faces) and not generic object categorization, indicating that these signatures arise from optimization for fine-grained face recognition. Furthermore, we recently found that functional segregation between face and object processing emerged spontaneously in a Dual-task CNN trained for both face identification and object categorization [25], similar to the functional organization seen in the brain. However, it remains unknown whether face pareidolia can be similarly replicated in CNNs and what specific task optimizations are necessary. Thus, by leveraging this approach, we aim to determine which types of visual experience and task optimization most closely mirror the behavioral and neural signatures of human face pareidolia processing.

Here, we used CNNs to explore how face pareidolia might arise in human perception. We hypothesized that this phenomenon could result from a combination of optimization for generic object categorization, which has previously been reported to result in coarse face detection [26,27], and fine-grained face identification. To test our hypothesis, we trained five CNNs, all based on the VGG16 architecture, on distinct tasks: (1) both object categorization and face identification (recognizing individual faces), (2) object categorization only, (3) face identification only, (4) object categorization and face detection (identifying the presence of any face), and (5) face identification and object detection (detecting the presence of an object). Notably, none of these networks was previously trained on pareidolia stimuli or designed with inherent face-specific biases. We then compared each CNN with human neural responses from a face pareidolia study [12], aiming to assess how closely the networks replicate human perceptual processing. In their study, Wardle et al. [12] recorded brain responses via magnetoencephalography (MEG) while participants viewed images of real faces, illusory faces (pareidolia), and matched objects. Using representational similarity analysis (RSA), we assessed the similarities in representation between the CNNs and MEG responses. Building on this, through model-based RSA, we probed how task optimization affects the layer-wise representational similarities of pareidolia stimuli in the CNNs. Finally, we investigated whether the CNNs rely on features similar to those humans use when classifying pareidolia stimuli as faces.

## Results

### Neural processing dynamics of pareidolia faces revealed by RSA

**Fig 1:**
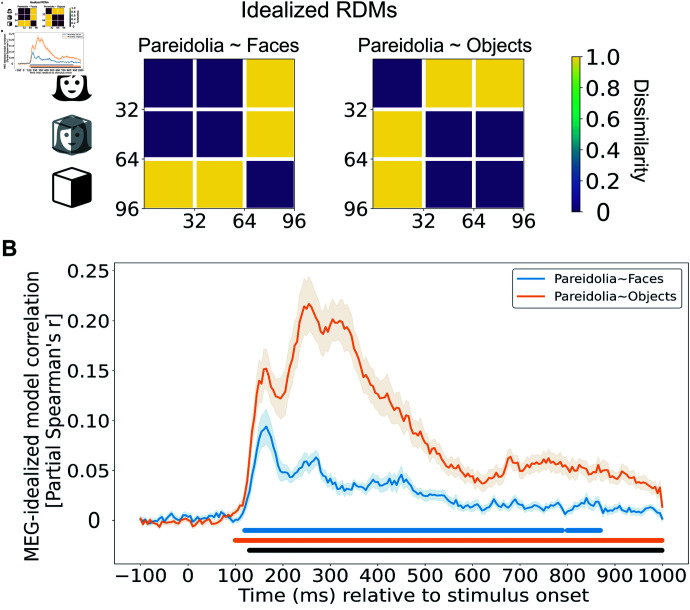
Neural correlates of pareidolia with the idealized models measured using RSA. A Idealized model RDMs reflecting two hypotheses on the representations of face pareidolia: i) ‘Pareidolia  ~  Faces’ hypothesis posits that pareidolia faces are represented similarly to real faces but differ from matched objects (left), and ii) ‘Pareidolia  ~  Objects’ hypothesis suggests that pareidolia faces are represented similarly to objects but differ from faces (right). The icons used in this figure have been obtained from The Noun Project (https://thenounproject.com/) under a royalty-free license. B Partial correlations between all time points of the neural MEG data and each idealized model RDM, controlling for the other model RDM. Partial correlations involve measuring the unique variance in the neural data explained by each idealized model, independent of the variance explained by the other model. These correlations reveal that the ‘Pareidolia  ~  Faces’ model uniquely explains variance in the neural data at early time points, indicating that pareidolia faces are initially represented similarly to real faces (peak at 165 ms after stimulus onset; blue line). However, the ‘Pareidolia  ~  Objects’ model uniquely explains increasing variance at later time points, suggesting that pareidolia faces progressively resemble matched objects and become segregated from real faces (peak at 255 ms after stimulus onset; orange line). Throughout the MEG time course, the partial correlation values for the ‘Pareidolia  ~  Objects’ model (orange line) are generally higher than those for the ‘Pareidolia  ~  Faces’ model (blue line), indicating that pareidolia faces are represented more similarly to objects than to real faces overall. Shaded areas indicate SEMs. Horizontal lines (in blue and orange) indicate time points of significant partial correlations (p  <  0.05) for each hypothesis, as determined by permutation clustering. The black line denotes time points of significant differences between the models.

How do neural representations of pareidolia faces, real faces, and matched objects evolve over time? To explore this question, we applied model-based RSA to neural MEG responses reported by Wardle et al. [12]. In their study, the authors presented participants (N = 22) with a validated stimulus set comprising 32 images each of real faces, pareidolia faces, and matched objects, measuring neural responses using MEG. They constructed representational dissimilarity matrices (RDMs) that captured pairwise dissimilarities between neural responses to each stimulus, sampled every 5 ms from 100 ms before to 1000 ms after stimulus onset. Building upon this method, we aimed to quantify the neural correlates of face pareidolia with matched objects and real faces. We first generated idealized model RDMs representing two hypotheses: i) pareidolia faces are represented similarly to real faces and differently from objects (‘Pareidolia  ~  Faces’ hypothesis; Fig 1A left), and ii) pareidolia faces are represented similarly to objects and differently from real faces (‘Pareidolia  ~  Objects’ hypothesis; Fig 1A right). Model-based RSA involves comparing these idealized RDMs with empirical neural data to test specific hypotheses about representational structures in the brain. We then computed partial Spearman correlations between the MEG RDMs and each idealized model RDM over time, controlling for the influence of the alternative model. This approach allowed us to assess the unique contribution of each hypothesis to the neural representations.

We observed that both model RDMs significantly correlated with the MEG data, yet they exhibited peak correlations at different time points. Initially, pareidolia faces were represented similarly to real faces, with the peak similarity occurring at 165 ms (max. correlation with ‘Pareidolia  ~  Faces’ model at 165 ms: r = 0.08; Fig 1B in blue). Concurrently, pareidolia faces also showed similar representations to objects, but this similarity reached its peak later at 255 ms (max. correlation with ‘Pareidolia  ~  Objects’ model at 255 ms: r = 0.20; Fig 1B in orange). Both hypotheses showed significant correlations with the MEG data from early processing onward (‘Pareidolia  ~  Objects’ from 100 ms to 1000 ms; ‘Pareidolia  ~  Faces’ from 120 ms to 790 ms and again from 800 ms to 870 ms post-stimulus; cluster-based permutation test, p  <  0.05). However, the representations began to diverge from 130 ms onward, with a correlation difference observed from 130 to 1000 ms (p  <  0.05). Notably, pareidolia faces were consistently more similar to objects than to faces throughout this period, with the most significant difference at 255 ms (max. difference at 255 ms: r = 0.15; Fig 1B in black). Collectively, these results corroborate the conclusions by Wardle et al. [12], suggesting an initial coarse detection stage around 165 ms, where pareidolia faces are represented more similar to faces than objects are. This is followed by a rapid transition between 165 and 255 ms, during which pareidolia faces and objects are increasingly represented more similarly, while real faces segregate from both.

### Task-optimization in CNNs affects alignment with neural MEG data in face pareidolia processing

Can the representations of pareidolia faces be explained by optimization for face identification and object categorization? We hypothesized that the phenomenon of initial similarity between pareidolia and real faces emerges from simultaneous optimization for object categorization and fine-grained face identification. To test this hypothesis, we used a previously developed dual-task network, based on the VGG16 architecture, that spontaneously segregated face and object processing into distinct processing systems [25]. This optimization enabled the network to learn distinctions between faces and objects (face detection), as well as among individual faces (face identification). However, is optimization for fine-grained face identification indeed necessary for the emergence of face pareidolia, or would a CNN without such face-specific training display similar representations of face pareidolia? Moreover, is face detection or object categorization training necessary for representational similarity between pareidolia and real faces to emerge? To probe these questions, we tested four additional CNNs, all based on the VGG16 architecture, each optimized for distinct tasks to determine the specific training conditions necessary for the emergence of face pareidolia: one trained exclusively on object categorization (Object-categorization CNN), one trained solely on face identification (Face-identification CNN), one trained on object categorization and face detection (i.e., with all face images assigned to a single ‘face’ category; Object-categorization-and-Face-detection CNN) and one trained on face identification and object detection (i.e., with all object images assigned to a single ‘object’ category; Face-identification-and-Object-detection CNN). Importantly, all networks were trained using the same training set as the Dual-task CNN, but either using solely the object images (i.e., Object-categorization CNN), solely the face images (Face-identification CNN), or both face and object images—with all face images (i.e., Object-categorization-and-Face-detection CNN) or object images (i.e., Face-identification-and-Object-detection CNN) assigned to a single category. We then evaluated these CNNs to determine how their training impacted their representations of pareidolia faces and their alignment with neural responses.

**Fig 2:**
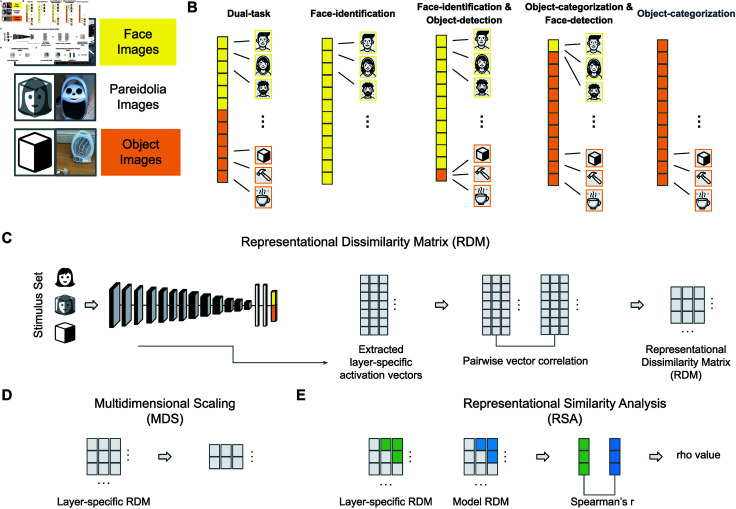
Experimental methods and analyses. A We compared the neural MEG responses with representations in CNNs using the 96 images from Wardle et al. [12]. The figure displays iconic placeholders representing the three different stimulus categories (i.e., faces, pareidolia, and matched objects), along with an example image used in the experiments. The icons used in this figure have been obtained from The Noun Project (https://thenounproject.com/) under the royalty-free license. Note that we do not have the right to display the human face images used in the experiments. The face image example shown in this figure is a similar photograph taken of one of the authors, who has granted permission for the publication of his identifiable image. The pareidolia and object images were sourced from Wardle et al. [12] (https://www.nature.com/articles/s41467-020-18325-8) and are used under a Creative Commons Attribution 4.0 International License (http://creativecommons.org/licenses/by/4.0/). For legal compliance, parts of the images containing logos or brands have been covered with a white box. All stimuli used in the experiments are available at the Open Science Framework (https://osf.io/9g4rz). B We used five task-optimized CNNs based on the VGG16 architecture, each trained on different combinations of face and object tasks: 1. Dual-task CNN: trained on face identification and object categorization; 2. Face-identification CNN: trained solely on face identification; 3. Face-identification-and-Object-detection CNN: trained on face identification and object detection; 4. Object-categorization-and-Face-detection CNN: trained on object categorization and face detection; and 5. Object-categorization CNN: trained solely on object categorization. C To generate a representational dissimilarity matrix (RDM), we initially passed the stimulus set through each CNN and extracted activations from specific layers of the network to obtain feature vectors. We then computed pairwise correlations between all feature vectors and subtracted them from 1, resulting in layer-wise RDMs. D We used multidimensional scaling (MDS) to visualize the obtained layer-wise RDMs. E By using representational similarity analysis (RSA), we measured the similarity (i.e., Spearman correlation between the upper triangles of the RDMs) between the layer-wise RDMs and the RDMs derived from different time steps of neural MEG data and idealized model RDMs.

Which task-optimized CNN would best align with the neural representations of pareidolia faces observed in the MEG responses? To find out, we directly compared each CNN with the neural MEG responses over time using RSA (Fig 2). For each CNN, we extracted activation patterns from the penultimate layer, applying the same 96 test stimuli that included real faces, pareidolia faces, and matched objects. We then calculated the pairwise distance (i.e., 1-correlation) between the response patterns of all units within a layer for each image. This resulted in one RDM for each CNN which we correlated with the neural MEG RDMs at all time points (-100 ms up to +1000 ms post-stimulus).

**Fig 3:**
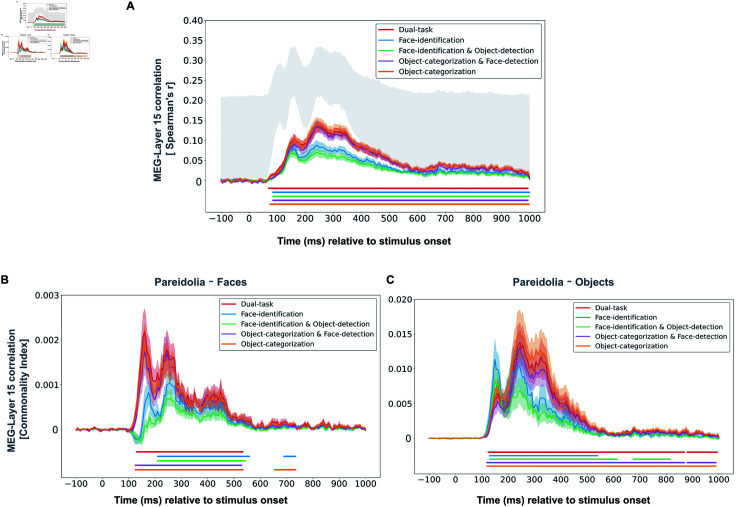
RSA between MEG responses and task-optimized CNNs across time. A Correlations between all time points of the neural MEG responses and the penultimate layer (layer-15) of each task-optimized CNN. CNNs including optimization for object categorization (i.e., Dual-task CNN, Object-categorization CNN, and Object-categorization-and-Face-detection CNN) showed a higher correlation than CNNs lacking optimization for object categorization (i.e., Face-identification CNN and Face-identification-and-Object-detection CNN). We have included the same analysis for all CNNs and layers in the Supplementary (see Supporting information). B Commonality index representing the unique variance shared between MEG data, task-optimized CNN, and idealized model RDM representing the similarity between pareidolia and faces (Pareidolia  ~  Faces model RDM), while controlling for the other model RDM (Pareidolia  ~  Objects model RDM). The similarity between pareidolia and faces contributes most to the correlation between the Dual-task CNN and the MEG data, followed by the Object-categorization and the Object-categorization-and-Face-detection CNN. C Commonality index representing the unique variance shared between MEG data, task-optimized CNN, and the idealized model RDM representing the similarity between pareidolia faces and objects (Pareidolia  ~  Objects model RDM), while controlling for the other model RDM (Pareidolia  ~  Faces model RDM). At early time points the similarity between pareidolia and objects contributes most to the correlation between the Face-identification CNN and the MEG data, while at later time points it mostly contributes to the correlation between the Object-categorization, followed by the Dual-task CNN and the Object-categorization-and-Face-detection CNN. Note the different scales used for B and C indicating that the commonality index of the ‘Pareidolia  ~  Faces’ model is substantially lower than that of the ‘Pareidolia  ~  Objects’ model. Horizontal lines below each plot indicate periods of significant correlation (cluster-based permutation test, p  <  0.05) for each CNN.

We found that all task-optimized CNNs significantly correlated with the MEG data across time, but they differed in the magnitude of correlation (Fig 3A). The Dual-task CNN showed a peak correlation of r = 0.13 (70 ms to 995 ms post-stimulus; cluster-based permutation test, p  <  0.05), the Object-categorization CNN had a peak correlation of r = 0.14 (75 ms to 1000 ms post-stimulus; cluster-based permutation test, p  <  0.05) and the Object-categorization-and-Face-detection CNN exhibited a peak correlation of r = 0.13 (85 ms to 995 ms post-stimulus; cluster-based permutation test, p  <  0.05), all peaking at 245 ms. These three CNNs, which all included object categorization in their task optimization, demonstrated a highly similar correlation time course with the MEG responses. In contrast, the correlations for the Face-identification CNN and the Face-identification-and-Object-detection CNN were weaker. The Face-identification CNN had a peak correlation of r = 0.08 (85 ms to 1000 ms post-stimulus; cluster-based permutation test, p  <  0.05), and the Face-identification-and-Object-detection CNN had a peak correlation of r = 0.07 (85 ms to 1000 ms post-stimulus; cluster-based permutation test, p  <  0.05), both at 240 ms. Overall, optimization for object categorization, as included in the Dual-task CNN, the Object-categorization CNN, and the Object-categorization-and-Face-detection CNN, resulted in better alignment with the MEG data compared to CNNs lacking this optimization. Perhaps unsurprisingly, this suggests that optimization for object categorization might play an important role in the neural representations of pareidolia faces, matched objects, and real faces. Contrary to our initial hypothesis, additional optimization for face identification in the Dual-task CNN did not further enhance alignment with neural responses. It is important to note that this analysis encompasses the entire representational similarity of all 96 stimuli, not solely focusing on pareidolia processing. Therefore, significant correlations might not exclusively reflect representational similarity with respect to pareidolia faces but could also indicate a more accurate representation of the other stimulus categories (e.g., within faces). Moreover, a significant correlation between CNN and MEG responses could reflect a similarity in representing pareidolia close to faces (‘Pareidolia  ~  Faces’ hypothesis), or an overlap in representing pareidolia close to objects (‘Pareidolia  ~  Objects’ hypothesis), as both hypotheses contributed to the MEG responses across time. These findings prompt further investigation into how specific task optimizations influence the alignment between CNNs and neural responses in representing pareidolia faces compared to real faces and objects.

To determine the specific contribution of each idealized model RDM to the alignment between task-optimized CNNs and the MEG responses, we performed a model-based commonality analysis (see Methods). Briefly, this approach quantifies the amount of variance uniquely shared among the MEG responses, a specific CNN, and each of the two idealized model RDMs (e.g., the ‘Pareidolia  ~  Faces’ hypothesis), termed the commonality index. This commonality index allows us to assess the unique contribution of each hypothesis to the alignment between CNNs and human neural responses.

The commonality analysis revealed distinct patterns among the task-optimized CNNs (Fig 3B and 3C). Specifically, the similarity between pareidolia faces and real faces (‘Pareidolia  ~  Faces’; Fig 3B) contributed most to the shared variance between the Dual-task CNN and the MEG responses, with a peak commonality index of 0.0021 at 160 ms (from 130 ms to 535 ms post-stimulus; cluster-based permutation test, p  <  0.05). This was followed by the Object-Categorization CNN (peak commonality index = 0.0018) at the same peak time (160 ms; from 125 ms to 535 ms post-stimulus), and the Object-categorization-and-Face-detection CNN (r = 0.0017) also peaking at 160 ms (from 125 ms to 530 ms post-stimulus). In contrast, the representational similarity between pareidolia and real faces contributed less to the shared variance in the Face-identification CNN (peak commonality index = 0.0010 at 255 ms; from 210 ms to 560 ms post-stimulus) and the Face-identification-and-Object-detection CNN (peak commonality index = 0.0007 at 260 ms; from 210 ms to 545 ms post-stimulus; all p  <  0.05).

Interestingly, the commonality analysis revealed a distinct pattern for the similarity between pareidolia faces and objects (‘Pareidolia  ~  Objects’) during the early processing time points (Fig 3C). This similarity contributed most to the shared variance between the Face-identification CNN and the MEG responses, with a peak commonality index of 0.011 at 150 ms (130 ms to 170 ms post-stimulus), and the Face-identification-and-Object-detection CNN, with a peak commonality index of 0.008 at 150 ms (130 ms to 170 ms post-stimulus). In contrast, this idealized model contributed much less to the Object-categorization CNN (peak commonality index of 0.007 at 160 ms), the Dual-task CNN (peak commonality index of 0.006 at 160 ms), and the Object-categorization-and-Face-detection CNN (peak commonality index = 0.005) during these early stages. These patterns transformed at later processing stages, where the similarity between pareidolia faces and objects (‘Pareidolia  ~  Objects’) contributed most to the shared variance between MEG responses and the Object-categorization CNN, with a peak commonality index of 0.015 at 245 ms (170 ms to 990 ms post-stimulus). The Dual-task CNN followed with a peak commonality index of 0.013 at 245 ms (170 ms to 870 ms post-stimulus), and the Object-categorization-and-Face-detection CNN matched this peak with a commonality index of 0.013 at 245 ms (170 ms to 870 ms post-stimulus). At these later stages, the similarity between pareidolia faces and objects contributed less to the shared variance between MEG responses and the Face-identification CNN (peak commonality index of 0.010 at 240 ms; 170 ms to 540 ms post-stimulus) and the Face-identification-and-Object-detection CNN (peak commonality index of 0.006 at 240 ms; 170 ms to 615 ms post-stimulus; all p  <  0.05).

These results suggest that task optimization for object categorization plays a critical role in enhancing the representation of pareidolia faces as real faces. This optimization enables the CNNs to more closely align with the neural representations of pareidolia in the MEG responses. However, it is important to note that the commonality index values for the ‘Pareidolia  ~  Faces’ model are much lower than those for the ‘Pareidolia  ~  Objects’ model, consistent with the stronger correlation observed between the ‘Pareidolia  ~  Objects’ model and the MEG responses. This warrants caution in interpreting these results, as the lower commonality index suggests that the ‘Pareidolia  ~  Faces’ model contributes less to the alignment compared to the ‘Pareidolia  ~  Objects’ model. Overall, our analysis suggests that task optimization differentially influences the representation of pareidolia stimuli and their relational proximity to objects and real faces. This differential influence mediates the alignment between task-optimized CNNs and MEG responses.

### Distinct representational patterns of face pareidolia across layers in task-optimized CNNs

To gain a better understanding of how task-optimization affects pareidolia representations across different layers in CNNs, we applied the same model-based RSA analysis used for the MEG data (Fig 2) to the task-optimized CNNs. For each CNN, we extracted layer-wise activation patterns in response to the 96 stimuli. This approach yielded an RDM for each CNN layer, reflecting the representational similarities of the stimuli. Using partial correlations, we measured which idealized model RDM (i.e., the hypothesis regarding the representation of face pareidolia; Fig 1A) best explained the activation patterns in each layer across the five task-optimized CNNs.

**Fig 4:**
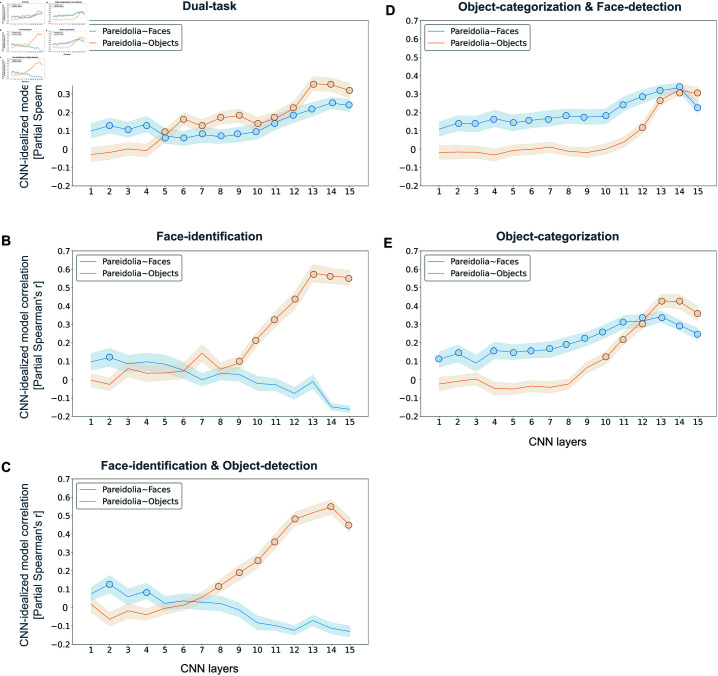
Effects of task optimization on pareidolia representations in CNNs. We used representational similarity analysis (RSA) to investigate how task optimization influences the representation of pareidolia faces, real faces, and matched objects in CNNs. For each CNN, we computed layer-wise representational dissimilarity matrices (RDMs) and calculated partial correlations with the two idealized models: ‘Pareidolia  ~  Faces’ (blue lines) and ‘Pareidolia  ~  Objects’ (orange lines). Partial correlations involve correlating each CNN RDM with one idealized model while controlling for the other, isolating the unique contribution of each model. Panels A - E displays the results for each CNN: A: Dual-task CNN (trained on face identification and object categorization), B: Face-identification CNN, C: Face-identification-and-Object-detection CNN, D: Object-categorization-and-Face-detection CNN, E: Object-categorization CNN. In CNNs that included object categorization training (Panels A, D, E), the representational similarity between pareidolia faces and real faces (‘Pareidolia  ~  Faces’; blue lines) increased across layers, indicating that these networks progressively represent pareidolia faces more like real faces in higher layers. Conversely, in CNNs lacking object categorization training (Panels B, C), this similarity was weak, only reaching significance at early layers, and decreased across layers. The Dual-task CNN showed a unique representational pattern where pareidolia faces were represented closer to matched objects than to real faces (‘Pareidolia  ~  Objects’; orange line), while still being more similar to real faces than objects (‘Pareidolia  ~  Faces’; blue line) from mid-layers (layer 5) onward, matching pareidolia representations in MEG (c.f. Fig 1). A multidimensional scaling (MDS) analysis of the Dual-task CNN is provided in the Supplementary Materials (see Supporting information). The shaded areas represent the standard error of the mean (SEM), bootstrapped across images. Colored circles indicate layers with significant correlations, determined using two-sided permutation tests with Bonferroni correction (p  <  0.05).

In the early layers of the Dual-task CNN (layers 1–4), we observed that pareidolia faces were represented more similarly to real faces than to objects (‘Pareidolia  ~  Faces’; Fig 4A in blue), with the maximum correlation occurring in layer 4 (r = 0.14). Beginning from layer 5, the representations underwent a reorganization. Pareidolia faces started to be represented more similarly to objects (‘Pareidolia  ~  Objects’; Fig 4A in orange), reaching the highest correlation in layer 13 (r = 0.36). Concurrently, the similarity between pareidolia and real faces further increased (max. correlation layer 14: r = 0.25), but remained lower than the similarity between pareidolia and objects. These results indicate that optimization for face and object recognition results in a processing dynamic where pareidolia faces are shifted toward real faces, but are overall more similar to objects than to faces.

In the Object-categorization CNN, we found that pareidolia faces and real faces were represented similarly until the late layers (layers 1–11) with a maximum correlation with ‘Pareidolia  ~  Faces’ in layer 13 (r = 0.31; see Fig 4E). Pareidolia faces started to show a greater similarity to objects than to faces only in the final network layers (layers 13–15; see Fig 4E), reaching a maximum correlation with ‘Pareidolia  ~  Objects’ in layer 13 (r = 0.43; see Fig 4E). This suggests that the transformation stage, at which pareidolia faces are processed as objects, emerged later and was less pronounced in a CNN without explicit face training, despite the presence of faces in numerous images within the object classes [28].

Consistent with the Object-categorization CNN, the Object-categorization-and-Face-detection CNN displayed a continuous representation of pareidolia as real faces across all layers (partial Spearman’s r  >  0.11 across layers; see Fig 4D), except for the last layer. Interestingly, real faces segregated less strongly from pareidolia faces and objects than in the other CNNs (max. partial Spearman’s r = 0.32 in layer 14). This occurred despite the network’s explicit training on (real) face detection. Thus, training for face detection seemed to result in a coarse face detection mechanism, including face pareidolia.

In the Face-identification CNN, the stage where pareidolia faces are represented similarly to real faces only weakly manifested in the first layers (layer 1–5) with a maximum correlation with ‘Pareidolia  ~  Faces’ in layer 2 (r = 0.12; see Fig 4B). This correlation was lower (partial Spearman’s r  <  0.12 across all layers) than that observed for the other CNNs. From the mid-level layers onwards, pareidolia faces were progressively represented more similarly to objects, and both pareidolia faces and matched objects strongly segregated from real faces (layer 7–15) with a maximum correlation with ‘Pareidolia  ~  Objects’ in layer 13 (r = 0.58; see Fig 4B). In the later layers, this CNN showed the most pronounced segregation of real faces from all other objects, suggesting that exclusive training on face discrimination results in the detection of real faces while making the CNN less susceptible to representing pareidolia faces as real faces. This finding might be due to the training diet being limited to real human faces, thereby affecting the network’s ability to generalize to object features. Consistent with this hypothesis, in prior work, we found that the features optimized for face identification are less useful for object categorization [25].

For Face-identification-and-Object-detection CNN, the overall pattern was very similar to the Face-identification CNN. However, it is important to note some subtle differences. The crossover point, where pareidolia faces begin to be more similarly represented to objects than to real faces, shifted from layers 6 to 7. The maximum correlation for ‘Pareidolia  ~  Faces’ in layer 2 slightly increased (r = 0.13 vs. r = 0.12 in the Face-identification CNN), while the peak correlation for ‘Pareidolia  ~  Objects’ decreased in layer 14 (r = 0.55 vs. r = 0.58 in the Face-identification CNN). These changes could be due to the inclusion of an explicit object category in the training regime of the Face-identification-and-Object-detection CNN, which enables the network to better differentiate pareidolia from objects, resulting in pareidolia being more closely represented to faces.

Together, these results suggest that task optimization and training diet significantly influence how pareidolia faces are represented within CNNs. CNNs lacking explicit training in fine-grained face discrimination tend to represent pareidolia faces more similarly to real faces initially, with a transition to object-like representation occurring only in the final processing stages. This suggests that fine-grained training in face recognition is crucial for rapidly segregating faces from objects and pareidolia. In contrast, CNNs trained predominantly for face recognition tend to minimally represent pareidolia faces similarly to real faces, highlighting the importance of training in object categorization for the networks to effectively detect faces in objects. Among the CNNs, the Dual-task CNN showed a more balanced representation, showing increasing correlations with both idealized models across layers. This suggests that optimization for both face identification and object categorization results in a processing dynamic where pareidolia faces gradually diverge from matched objects, yet remain more similar to objects than to faces.

When comparing the representational patterns in CNNs with those observed in the MEG responses (refer to Fig 1B), several notable differences emerge. Firstly, in the early layers of all CNNs, pareidolia faces are initially represented more similarly to real faces than to objects. This specific pattern does not appear in the MEG data. However, it is important to note that the significant difference between the ‘Pareidolia  ~  Objects’ and the ‘Pareidolia  ~  Faces’ models in the MEG data only emerges at 130 ms post-stimulus. Thus, the early processing layers may reflect neural processing at time points before 130 ms. Secondly, an increasing correlation with both models, while pareidolia stimuli are consistently more similar to objects than to faces from early stages onward, was observed uniquely in the Dual-task CNN. However, the magnitude of difference between the two models in the Dual-task CNN is much smaller and less pronounced than was observed in the MEG data. Lastly, unlike the MEG data where the correlations with both idealized models peak at different processing stages, the CNNs show a positive correlation of ‘Pareidolia  ~  Objects’ in late layers (i.e., Dual-task CNN, Object-categorization CNN, and Object-categorization-and-Face-detection CNN), the peaks occur at similar layers (around layers 13/14). This discrepancy could be attributed to neural feedback processes, which are likely involved in the second peak of the ‘Pareidolia  ~  Objects’ model at 255 ms in the MEG data but are not captured by the feed-forward architecture of the CNNs.

### Dual-task CNN exploits face-like features for pareidolia stimuli classification as faces

**Fig 5:**
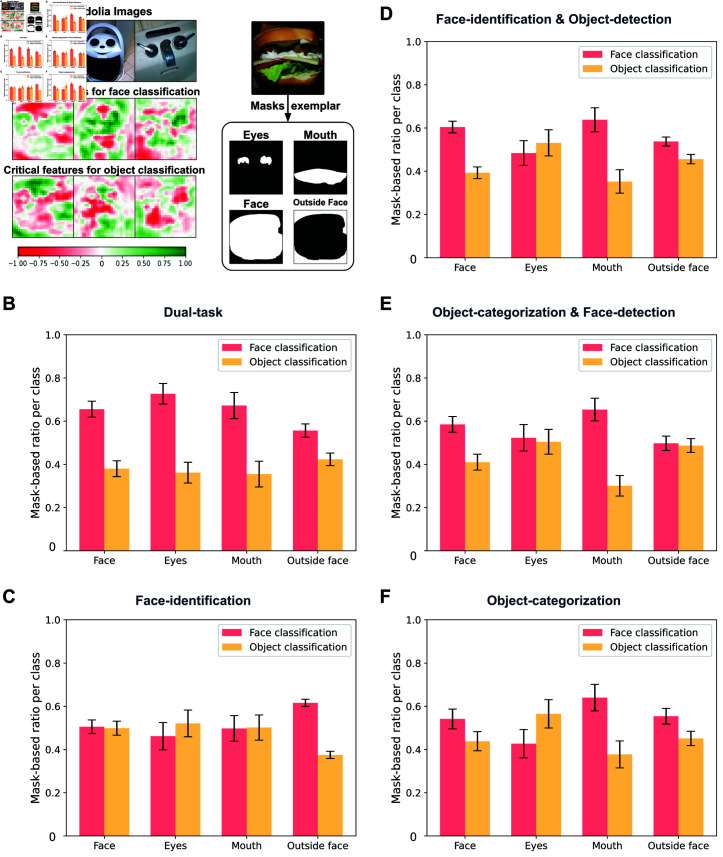
Visualization of critical features used by the Dual-task CNN to classify face pareidolia stimuli. A Visualization of the critical features in sample pareidolia stimuli (top row) that the Dual-task CNN uses to classify these stimuli as either ‘face’ (middle row) or ‘object’ (bottom row). Green areas indicate positive class attribution, essential for classification, while red areas signify negative class attribution, detrimental for classification. The pareidolia images were sourced from Wardle et al. [12] (https://www.nature.com/articles/s41467-020-18325-8) and are used under a Creative Commons Attribution 4.0 International License (http://creativecommons.org/licenses/by/4.0/). Parts of the object and pareidolia images containing logos or brands have been covered with a white box for legal compliance. Example masks are used to quantify the use of face-like features in classifying pareidolia stimuli as ‘face’ or ‘object’. For each pareidolia stimulus, we generated masks corresponding to the entire face (‘Face’), specific face features like eyes (‘Eyes’) and mouth (‘Mouth’), and the area outside the face (‘Outside Face’). We calculated the mean pixel ratio within each mask (ranging from 0, indicating the lowest, to 1, the highest) for classifying the stimulus as ‘face’ (red bars) or as ‘object’ (yellow bars). B, C, D, E, F This mask-based ratio is calculated for all the CNNs. The analysis revealed that the Dual-task CNN primarily relies on facial features, especially the eye region, to classify the pareidolia stimulus as a face. Areas outside the face were similarly used in both classifications. Such clarity is not observed in other CNNs. Error bars denote SEM across stimuli.

Pareidolia face stimuli exhibit object features with face-like characteristics, which humans rely on when perceiving pareidolia stimuli [29]. This leads us to hypothesize that CNNs demonstrating this phenomenon also use these features to represent pareidolia stimuli similarly to real faces. However, CNNs may employ distinctive strategies compared to humans [30]. To investigate the critical features used by CNNs for classifying pareidolia stimuli as faces, we applied advanced visualization and interpretability techniques to each task-optimized CNN. Specifically, we generated saliency maps for pareidolia stimuli using an occlusion-based algorithm that evaluates the contribution of each pixel to the classification [31]. This allowed us to identify the crucial features in pareidolia stimuli that a CNN relies on to categorize them as faces or objects. Fig 5A displays examples of these saliency maps generated for the Dual-task CNN overlaid on the original pareidolia stimuli. In these maps, green areas indicate the critical features necessary for classifying the pareidolia image (top row) as either a face (middle row) or an object (bottom row). These saliency maps suggest that the Dual-task CNN primarily used face-like object features, such as ‘eyes’, ‘nose’, and ‘mouth’, to classify a pareidolia stimulus as a face. However, can we objectively quantify this observation, and do the other task optimizations lead to similar processing mechanisms?

To further quantify this observation, we created specific masks for the 32 pareidolia stimuli images (Fig 5A). These masks correspond to various face-like features, such as eyes and mouth, the entire face, and areas outside the face. We then calculated the proportion of critical pixels within each mask that contributed to classifying the stimulus as either a ‘face’ or an ‘object’, averaging these proportions across all stimuli. We performed this analysis for all CNNs (Fig 5B). Our analysis revealed that the Dual-task CNN strongly relies on facial features (mean pixel ratio within the ‘Face’ area: 0.65), particularly the eyes (0.72), followed by the mouth (0.67), for face classification. These ratios were much lower when the CNN classified images as objects. Interestingly, the CNN used areas outside the face with approximately equal emphasis for both ‘face’ and ‘object’ classifications. The other four CNNs showed noticeable differences. The Face-identification CNN did not show any preference for face-like features when classifying pareidolia stimuli as faces, showing similar reliance for both classifications on the eyes (mean pixel ratio within the ‘Eyes’ area for ‘faces’: 0.46 versus ‘objects’: 0.52), mouth (‘faces’: 0.49 versus ‘objects’: 0.50), and the entire face (‘faces’: 0.50 versus ‘objects’: 0.49). The Object-categorization, the Object-categorization-and-Face-detection, and the Face-identification-and-Object-detection CNN all showed larger ratios for the general face area (Object-categorization CNN: ‘faces’: 0.54 versus ‘objects’: 0.43; Object-categorization-and-Face-detection CNN: ‘faces’: 0.58 versus ‘objects’: 0.41; Face-identification-and-Object-detection CNN: ‘faces’: 0.60 versus ‘objects’: 0.39) and the mouth (Object-categorization CNN: ‘faces’: 0.64 versus ‘objects’: 0.37; Object-categorization-and-Face-detection CNN: ‘faces’: 0.65 versus ‘objects’: 0.30; Face-identification-and-Object-detection CNN: ‘faces’: 0.63 versus ‘objects’: 0.35) when classifying pareidolia stimuli as ‘faces’ compared to ‘objects’. However, none of them showed a difference in relying more on the eyes for the ‘face’ classification, with some of them even showing the opposite trend (Object-categorization CNN: ‘faces’: 0.42 versus ‘objects’: 0.56; Object-categorization-and-Face-detection CNN: ‘faces’: 0.52 versus ‘objects’: 0.50; Face-identification-and-Object-detection CNN: ‘faces’: 0.48 versus ‘objects’: 0.53). Previous work suggested that the eyes are most critical for humans when processing pareidolia stimuli [29]. These findings suggest that optimization for both face identification and object categorization, as found in the Dual-task CNN, is necessary to focus on face-like features in objects when detecting pareidolia stimuli as faces.

## Discussion

Our goal in this study was to investigate why the human visual system employs an initial coarse face detection mechanism, which can lead to the phenomenon of seeing faces in things. To this end, we compared the representations of face pareidolia in CNNs, with various experiences and task optimization for faces and objects, to neural MEG data. Our findings suggest that CNNs that include object categorization in their task optimization align more closely with neural data. This alignment was driven by both the initial similarity between pareidolia stimuli and faces, as well as the segregation of real faces from pareidolia and objects. We then analyzed the processing dynamics of pareidolia, matched objects and faces across layers in all CNNs. The Dual-task CNN exhibited an early coarse face detection mechanism, where pareidolia faces were represented more similarly to real faces than to matched objects. This representation was followed by a transformation, during which real faces became segregated from both objects and pareidolia faces. Importantly, CNNs optimized solely for face identification or face identification and object detection showed almost no evidence of pareidolia stimuli being represented similarly to real faces. In contrast, optimization for object categorization alone, or combined with face detection, was sufficient to capture this coarse face detection stage. However, unlike neural processing dynamics, real faces were segregated from objects and pareidolia only at the latest stages in these networks. Critically, an investigation of the crucial features used to classify pareidolia stimuli as ‘faces’ revealed that only the Dual-task CNN used features similar to those humans rely on, particularly the eyes and the mouth. Overall, these results suggest that the early coarse face detection mechanism in the human brain may arise from task-specific optimizations for both object and face recognition.

Our findings also provide some insights into potential neural mechanisms underlying the face detection mechanism in the brain. Previous research has suggested the existence of innate face templates that direct attention towards faces [32,33], possibly through subcortical mechanisms [34]. Despite the absence of explicit face-specific biases in the CNNs, our results indicate that the generic face detection mechanism observed in these networks was capable of representing pareidolia faces similarly to real faces. However, it is important to note that our comparison between CNN representations and MEG responses covered the full sensor set, which might not effectively capture or predominantly represent subcortical responses [35,36]. Future studies comparing CNNs with neural responses to face pareidolia in subcortical areas, such as the amygdala [37], could help further elucidate the contributions of cortical and subcortical processes to the detection of face pareidolia.

Moreover, previous studies have proposed that top-down processes, such as the social aspects of faces [38] or predictive top-down signals [39,40], contribute to the phenomenon of pareidolia. However, our findings, indicating that feed-forward CNNs that included object categorization in their training generally aligned with the neural representations of face pareidolia, suggest that such top-down processes may not be essential for recognizing faces in objects. One possible explanation for these contradictory findings is that studies reporting evidence of top-down processing often use ambiguous stimuli (e.g., contrast-modulated images, [39]) or noise stimuli (e.g., black-and-white noise stimuli [40]), where top-down processes might play a more prominent role. In contrast, our study used stimuli that resemble objects with face-like features, and our results imply that feed-forward processing of stimulus features in the CNNs was sufficient for the emergence of face pareidolia. Nevertheless, as our research was limited to feed-forward networks, which did not explain all variance, we cannot rule out that CNNs incorporating recurrent or top-down processes might provide a more accurate representation of neural responses to face pareidolia.

The observation that, in all CNNs, pareidolia faces are initially represented more similarly to real faces but later align more closely with objects provides evidence for the utilization of lower-level representational similarities in CNNs to organize visual information before higher-level distinctions based on unique categories or identities emerge. In a control analysis, we indeed found that the similarity between pareidolia and faces in the CNNs was reduced when controlling the stimuli for low-level features (see S3 Fig). These findings are consistent with recent research indicating a coarse-to-fine organization of the human face perception system [41,42]. By manipulating task optimization in CNNs, our findings also hint at the potential role of loss functions in explaining this organizational principle. Our results show that general exposure to objects is sufficient for developing a broad face detection mechanism. However, the optimization for face-specific visual experiences is crucial for segregating faces from objects and pareidolia at an early stage of visual processing. These findings are consistent with neuroscientific research, which has observed a broad-based face-processing mechanism in the brain that becomes refined and specialized for specific face identities through experience [43–46]. Overall, our results suggest that a coarse face detection mechanism, which becomes fine-tuned by face-specific experience, is a common characteristic shared by both biological and artificial networks. Nonetheless, it’s crucial to acknowledge that perceiving face pareidolia is a complex phenomenon, modulated by perceptual awareness [47], and a simple coarse-to-fine organizational framework may not fully encompass all facets of this process.

We would like to clarify that, within the scope of our study, the term ‘representation’ specifically denotes the pattern of neural activities captured by MEG, which correlates with distinct cognitive or perceptual processes. It is important to recognize and acknowledge the inherent limitations of MEG, such as its primary focus on cortical activities and its indirect approach to correlating neural activity with behavior. These limitations suggest that MEG offers a somewhat constrained perspective, largely centered on cortical dynamics. Consequently, any interpretations derived from MEG data should be contextualized within these bounds (c.f. [48]). To address these limitations, our study aims to enhance the interpretative value of our findings by linking CNNs not just to MEG data but also to behavioral signatures of face pareidolia processing [49].

In this study, we used deep neural networks to investigate how specific task optimizations contribute to the phenomenon of face pareidolia in humans. Deep neural networks serve as a valuable tool for expressing and testing computational hypotheses, as demonstrated by an increasing body of research in the field [18,50,51]. Our work represents a step towards developing a computational account of face pareidolia. However, several aspects of deep neural networks could further enhance our understanding of this phenomenon. Firstly, our analyses are based on all units in a specific CNN layer, and thus, we do not specifically address the role of individual units involved in the computations for processing faces and objects [25,52,53]. Investigating the contribution of individual category-selective units in the representation of pareidolia may provide additional insights into the processing of face pareidolia. Secondly, our current study does not encompass the potential impact of different types of learning algorithms and architectures that may better explain the neural data. Recurrent, unsupervised, or self-supervised neural networks are promising approaches in this regard [54–59]. Exploring these alternative networks could shed further light on the underlying mechanisms of face pareidolia. Lastly, the architecture and training algorithm we employed here does not account for the topographical organization of different category-selective regions, as identified in the ventral visual pathway. Recent research suggests that networks incorporating topographical constraints predict the functional organization of cortical areas in the visual system [60,61] and provide a better match for the dimensional structure of brain representations [62]. In the future, it would be interesting to investigate the representations of face pareidolia in such topographic networks and compare them to the neural responses observed in category-selective regions of the ventral visual pathway.

In conclusion, our work offers a first step toward a computational account for the emergence of face pareidolia in the human visual system, suggesting that this phenomenon may arise not as an isolated curiosity, but as a by-product of the system’s optimization for face and object recognition. By using CNNs optimized for various tasks related to face and object recognition and comparing them with neural MEG data, we found an enhanced similarity between the representations of face pareidolia in networks trained on object categorization and the human brain, compared to networks lacking this optimization. Critically, a detailed investigation of the processing mechanisms of face pareidolia in the CNNs revealed that only an optimization for both object categorization and face identification resulted in human-like processing. These findings underline the relevance of interpretability methods when comparing artificial neural networks to complex perceptual phenomena in humans. In light of these findings, we propose that further study of face pareidolia, its origins, and its representations within both the human brain and artificial systems, will provide invaluable insights not only into the mechanisms underlying face and object perception but also into the broader functioning of our visual system.

## Materials and methods

### Experimental stimulus set

We used a stimulus set provided by Wardle et al. [12] comprising 96 images sourced from the internet (Fig 2A). The stimulus set consisted of 32 pareidolia faces, 32 matched non-face objects, and 32 human faces. To ensure a meaningful comparison, the matching objects were carefully selected to belong to the same object category as the pareidolia faces found in objects (e.g., bell peppers, rucksack, coffee cup), while possessing similar visual characteristics. To match the high variability observed in the pareidolia face images, the human faces were deliberately chosen to exhibit high degrees of variance across age, facial expression, gender, race, and head position. Additionally, since two pareidolia images also feature two ‘faces’, two images in the human face set were selected to depict two individuals. Every image was cropped and resized to (400  ×  400) pixels. It is important to note that the stimuli were not adjusted to control for low-level visual differences (like luminance or contrast). We selected this particular set of stimuli because of the accessibility of the neural data and due to its previous application in behavioral and neural studies [63–65], which allows for a direct comparison with established research in the field.

### Neural MEG data

To compare the CNNs with representations obtained from different time points of the MEG data, we used neural data provided by Wardle et al. [12]. The neural data comprised representational dissimilarity matrices (RDMs) obtained from 100 ms before to 1000 ms after stimulus onset (in 5 ms increments) for each of the 22 participants. The RDMs were generated by correlating the whole-brain patterns across MEG sensors, after preprocessing, for every pair of the 96 stimuli. The 1 - Spearman’s r correlation coefficient served as the distance measure. Comprehensive information regarding the experimental procedure and MEG data preprocessing can be found in [12].

### Representational similarity analysis between idealized models and MEG data

To directly assess the neural representational dynamics of pareidolia faces, real faces, and objects in the MEG data, we used representational similarity analysis (RSA; [66]). We constructed two idealized RDMs to represent two distinct hypotheses: First, real faces and pareidolia faces are represented similarly but distinct from objects (‘Pareidolia  ~  Faces’; Fig 1A left). Second, pareidolia faces are represented similarly to matched objects but distinct from faces (‘Pareidolia  ~  Objects’; Fig 1A right). In these idealized RDMs, the low dissimilarity between image categories (e.g., between real faces and pareidolia in the ‘Pareidolia  ~  Faces’ model) is represented as 0, and high dissimilarity as 1 (e.g., between pareidolia faces and objects in the ‘Pareidolia  ~  Faces’ model).

To measure the presence of these hypotheses across neural processing, we computed correlations between each idealized model RDM and time-wise MEG RDMs. Specifically, for each time point, we computed the partial rank correlation (Spearman’s r) between the dissimilarities vector (upper triangle of the RDM) of each idealized RDM (while controlling for the other idealized RDM) and the corresponding MEG dissimilarities vector (upper triangle of the MEG RDM). We used a permutation-based cluster-size inference method for the statistical analysis of partial correlation time series, employing the MNE-Python toolbox [67]. In this approach, the null hypothesis assumes no correlation, meaning correlation values or differences are zero. The process for identifying significant temporal clusters involved several steps. Firstly, we randomized the condition labels in the MEG data by applying a sign permutation test, where subject responses were randomly multiplied by  + 1 or  - 1. This randomization was repeated 1,024 times, generating a permutation distribution for each time point. Subsequently, time points surpassing the 95th percentile of this distribution were considered cluster-inducing, indicating a significance level of p  <  0.05 (two-sided). Finally, we defined significant clusters in time as those exceeding the 95th percentile in terms of the number of contiguous time points that were significant, according to the permutation distribution, also reflecting a significance level of p  <  0.05 (two-sided).

### Task-optimized convolutional neural networks

To investigate the influence of task optimization on the emergence of face pareidolia, we used five distinct CNNs, each trained for a specific task (Fig 2B). All CNNs were based on the VGG16 architecture [68]. First, we used a Dual-task CNN to test whether face pareidolia would emerge in a network trained to detect and recognize faces amidst objects. This CNN was trained on face identity categories (1,714) from the VGGFace2 dataset [69] and object categories (423) selected from the ImageNet dataset [70]. It is important to note that the object dataset did not include a ‘face’ class. However, faces are still present in numerous images within the object classes [28]. The two tasks were combined in a single-task classification layer, resulting in a total of 2,137 categories. To achieve task performance, the CNN thus needed to discriminate faces from objects (i.e., face detection) and to discriminate faces from each other (i.e., face recognition). Additional details regarding the training and test sets can be found in the previous work by Dobs et al. 2022 [25]. Second, we used an Object-categorization CNN to investigate the representational effect of real faces, pareidolia faces, and matched objects. This network was optimized for generic object categorization and trained on the same 423 object categories as the Dual-task CNN. Third, to investigate whether optimization for face identification would be sufficient for face pareidolia to emerge, we included a Face-identification CNN trained exclusively on face identity identification. This CNN used the same 1,714 face identity categories as the Dual-task CNN. Fourth, we used an Object-categorization-and-Face-detection CNN, trained to categorize objects and detect faces (c.f. [24]). This CNN was trained on the same dataset as the Dual-task CNN, but all face images were assigned to a single ‘face’ class. Thus, this network shared a similar training experience with the Dual-task CNN but was not specifically optimized for fine-grained discrimination of faces. Fifth, we used a Face-identification-and-Object-detection CNN, trained to detect objects and identify faces. This CNN was trained on the same dataset as the Dual-task CNN, but all object images were assigned to a single ‘object’ class.

For the training of all CNNs, each image was scaled to a minimum side length (height or width) of 256 pixels, standardized to a mean and standard deviation of 0.5, and subjected to data augmentation techniques (i.e., 20% gray-scaling, random cropping to 224 × 224 pixels). The test images were scaled, normalized, and center-cropped before extracting the classification performance. The training parameters were similar to those proposed in [68]: stochastic gradient descent (SGD) optimization with momentum, an initial learning rate of 10^−3^, weight decay of 10^4^, and momentum of 0.9. The cross-entropy loss was calculated on random batches (batch size of 128 images), and back-propagation was used to update the weights during training. All networks were trained in Python 3.7 using the Pytorch deep learning framework on NVIDIA GPU clusters.

### Representational dissimilarity matrices of CNNs

To compare the representational effect of real faces, pareidolia faces, and matching objects in all CNNs with the neural data, we transformed the CNN layers into Representational Dissimilarity Matrices (RDMs) (Fig 2C). For each of the 13 convolutional layers and the first two fully connected layers (excluding the classification layer) in all CNNs, we extracted activation patterns separately for each of the 96 stimuli (32 face images, 32 object images, and 32 pareidolia images). These activation patterns were unrolled into vectors, and pairwise distances between the vectors were computed using correlation distance (1 – Pearson’s r). The resulting dissimilarities were then used to construct a 96 × 96 Representational Dissimilarity Matrix (RDM) per layer for each CNN. Additionally, smaller category-specific RDMs were obtained by averaging the dissimilarity values within each category (e.g., real faces) to obtain 3 × 3 RDMs. The diagonals were omitted when calculating the means for category-specific RDMs.

### Representational similarity analysis between CNNs and neural MEG data

To quantify the extent to which the CNNs correlated with neural representations, we used RSA (Fig 2E). We computed the correlation between the representational dissimilarities for the 96 stimuli in each layer in all CNNs and the time-wise MEG RDMs for each of the 22 participants. Specifically, we computed the rank correlation (Spearman’s r) between the dissimilarity vector obtained in the penultimate layer of each CNN (upper diagonal of the RDM) and the participant-wise dissimilarity vectors (upper diagonal of the RDMs) for all MEG time points. We also obtained a noise ceiling for each time point based on the variation in neural RDMs across participants [71]. We used a permutation-based cluster-size inference method for the statistical analysis of partial correlation time series, employing the MNE-Python toolbox [67]. In this approach, the null hypothesis assumes no correlation, meaning correlation values or differences are zero. The process for identifying significant temporal clusters involved several steps. Firstly, we randomized the condition labels in the MEG data by applying a sign permutation test, where subject responses were randomly multiplied by  + 1 or  - 1. This randomization was repeated 1,024 times, generating a permutation distribution for each time point. Subsequently, time points surpassing the 95th percentile of this distribution were considered cluster-inducing, indicating a significance level of p  <  0.05 (two-sided). Finally, we defined significant clusters in time as those exceeding the 95th percentile in terms of the number of contiguous time points that were significant, according to the permutation distribution, also reflecting a significance level of p  <  0.05 (two-sided).

### Commonality index between MEG data, task-optimized CNNs, and idealized model RDMs

To assess the unique shared variance between MEG data, task-optimized CNNs, and idealized model RDMs, we employ the commonality index [72]. Specifically, for the penultimate layer of each CNN, we calculate the partial rank correlation (Spearman’s r). This correlation is computed between the dissimilarity vectors (upper triangle of the RDM) of each neural data time point and the corresponding CNN dissimilarity vector (upper triangle of the layer-15 RDM) while controlling for the influence of one of the idealized RDMs. This procedure is then repeated, each time controlling for the other idealized RDM, and finally for both idealized RDMs together. The commonality index for a particular idealized model is determined by subtracting the squared correlation coefficient (when the other idealized RDM is controlled for) from the squared correlation coefficient when both idealized RDMs are controlled for. This calculation quantifies the unique contribution of each idealized model to the shared variance with the MEG data and CNN outputs.

### Representational similarity analysis between idealized models and CNNs

To directly assess the representational transformation of pareidolia faces, real faces, and objects across layers in the five different CNNs, we used RSA. We used the same two idealized RDMs as used for the MEG analysis. We used the previously described process for identifying significant temporal clusters in this analysis. To measure the presence of these hypotheses across CNN layers, we computed correlations between each idealized RDM and the layer-wise RDMs obtained from the Dual-task CNN. Specifically, for each layer, we computed the partial rank correlation (Spearman’s r) between the dissimilarities vector (upper triangle of the RDM) of each idealized RDM (while partialling out the other idealized RDM) and the corresponding CNN dissimilarities vector (upper triangle of the layer-specific RDM). To assess the statistical significance, we performed bootstrapping by randomly sampling stimuli (with replacement) and then computing the correlation with idealized RDMs and CNN RDMs 1,000 times. To avoid inflating the correlations, we excluded all nondiagonal pairs of identical images that arose due to sampling with replacement. The standard deviation of this bootstrapped distribution served as SEM. We have also included detailed significance testing using permutation tests for the correlations. The images were permuted 10,000 times, and the created RDMs were correlated with the model RDMs to form a null distribution. The p-value is determined using two-sided permutation tests with Bonferroni correction (p  <  0.05) by comparing the true correlation with the null distribution (greater or less than).

Furthermore, to investigate the role of task optimization in the representational transformations of pareidolia faces, real faces, and objects, we extended the same analysis to four additional CNNs: the Object-categorization CNN, the Face-identification CNN, Face-identification-and-Object-detection CNN, and the Objects-and-face categorization CNN (refer to the section ‘Task-optimized convolutional neural networks’ for details).

### Controlling low-level image properties

To assess the impact of low-level image properties on the representational similarities of pareidolia faces, we conducted a model-based RSA on the same 96 stimuli but matched for these properties (see S3 Fig). This approach allows us to determine if the observed representations are influenced by factors such as luminance and color, rather than higher-level perceptual or cognitive processes. For this purpose, we processed the 96 stimulus images using the SHINE_color MATLAB toolbox [73], which allows for precise control of luminance while maintaining color integrity. The images were adjusted in the HSV (Hue, Saturation, Value) color space, which is particularly suited for manipulations that require independent control of color and intensity. The toolbox used the specMatch and histMatch functions to perform one iteration over the entire image set, applying these adjustments based on the default settings of the color toolbox.

### Visualizing critical features in pareidolia stimuli used by task-optimized CNNs

To examine whether the task-optimized CNNs rely on face-like features to represent pareidolia faces as ‘faces’, we used network interpretability techniques. Specifically, we used an occlusion-based attribution method originally proposed by Zeiler et al. [31]. This method estimates the areas of an image that are critical for a classifier’s decision by occluding different regions of the image and quantifying the resulting change in classification. A sliding window with a predefined stride is applied to the image to generate a class-selective heatmap. In the resulting heatmaps, green areas highlight image features crucial for the classification of a specific class (e.g., face), while red areas depict features critical for the classification of the other class (e.g., object). For this analysis, we replaced the final classification layer in each CNN with a two-way classifier specifically designed for face and object classification. This modification preserved the rest of the network architecture. The new binary output network was fine-tuned by freezing all the convolutional layers and training only the fully connected layers. For fine-tuning, we used an independent dataset comprising 1,000 faces and 1,000 objects (100 categories each). The face dataset, sourced from VGGFace2, included 100 different identities (10 images each) not used in the original CNN training. The object dataset, selected from the Things dataset [74], comprised 100 categories (10 images each) distinct from those in the initial object training. We then processed the 32 pareidolia images through the updated CNNs to identify critical features.

To understand the role of face features in detail, we manually created masks for each of the 32 pareidolia stimuli. These masks included one for the entire face (‘Face’ mask), masks for specific facial features like eyes and mouth (‘Eyes’ and ‘Mouth’ masks), and a mask for the area surrounding the face (‘Outside Face’ mask). To determine the importance of these areas in classifying a stimulus as either a ‘face’ or an ‘object’, we calculated a ratio for each mask in each class-specific heat map. This ratio was derived by dividing the number of pixels with positive class attributions (values above zero) by the total number of pixels in the masked area. The resulting ratio, ranging from 0 to 1, indicates the importance of the masked area in classification, with 0 implying no significance and 1 indicating critical importance. We then averaged these ratios for each mask across all stimuli for both face and object classifications and computed the SEM across the stimuli.

## Supporting information

S1 Fig Representations of faces, pareidolia faces, and matched objects across all layers of the Dual-task CNN - Dual-task CNN layers 1–15. The Dual-task CNN’s representational dissimilarity matrices (RDMs) for the stimulus set of faces, pareidolia faces, and matched objects in all CNN layers are shown here. Dimensionality reduction of the RDMs using Multidimensional Scaling (MDS) is shown to the right of each RDM along with the category-specific RDMs constructed by averaging the corresponding larger matrix. The icons used in this figure have been obtained from The Noun Project (https://thenounproject.com/) under a royalty-free license.
(TIF)

S2 Fig Correlations of all CNN layers (Dual-task (A), Face-identification (B), Face-identification-and-Object-detection (C), Object-categorization-and-Face-detection (D), Object-categorization (E)) with neural MEG time course. Each panel shows the correlation between MEG data and neural network layers over time relative to stimulus onset. Green lines represent individual layers, with darker shades indicating deeper layers (15-darkest). The gray shaded area represents the noise ceiling. Horizontal lines below each plot indicate periods of significant correlation for each CNN.
(TIF)

S3 Fig Pareidolia representations in CNNs (Dual-task (A), Face-identification (B), Face-identification-and-Object-detection (C), Object-categorization-and-Face-detection (D), Object-categorization (E)) while controlling low-level image properties. To assess the impact of low-level visual differences, such as luminance and contrast, among the stimuli, we conducted control analyses using the SHINE toolbox [73]. This step was crucial to ensure that any observed differences in representations were not merely due to these low-level features. In these analyses, we matched the low-level features of the face, object, and pareidolia stimuli across the different models: For the ‘Pareidolia  ~  Objects’ model RDM (represented in orange), the representation remained robust even after adjusting for low-level features. Conversely, for the ‘Pareidolia  ~  Faces’ model RDM (represented in blue), the representation dipped substantially after controlling for these features. These findings indicate that low-level features in non-controlled images play a crucial role in driving the perception of face pareidolia across all CNNs. This underscores the importance of controlling for such variables to isolate higher-level cognitive processes involved in face and object recognition. The shaded areas represent the standard error of the mean (SEM), bootstrapped across images. Colored circles indicate layers with significant correlations, determined using two-sided permutation tests with Bonferroni correction (p  <  0.05).
(TIF)
