# Supplementary figures and images for: Human-like face pareidolia emerges in deep neural networks optimized for face and object recognition
Source: PLoS Comput Biol. 2025 Jan 27;21(1):e1012751. doi: 10.1371/journal.pcbi.1012751 (PMC11790231; doi:10.1371/journal.pcbi.1012751)

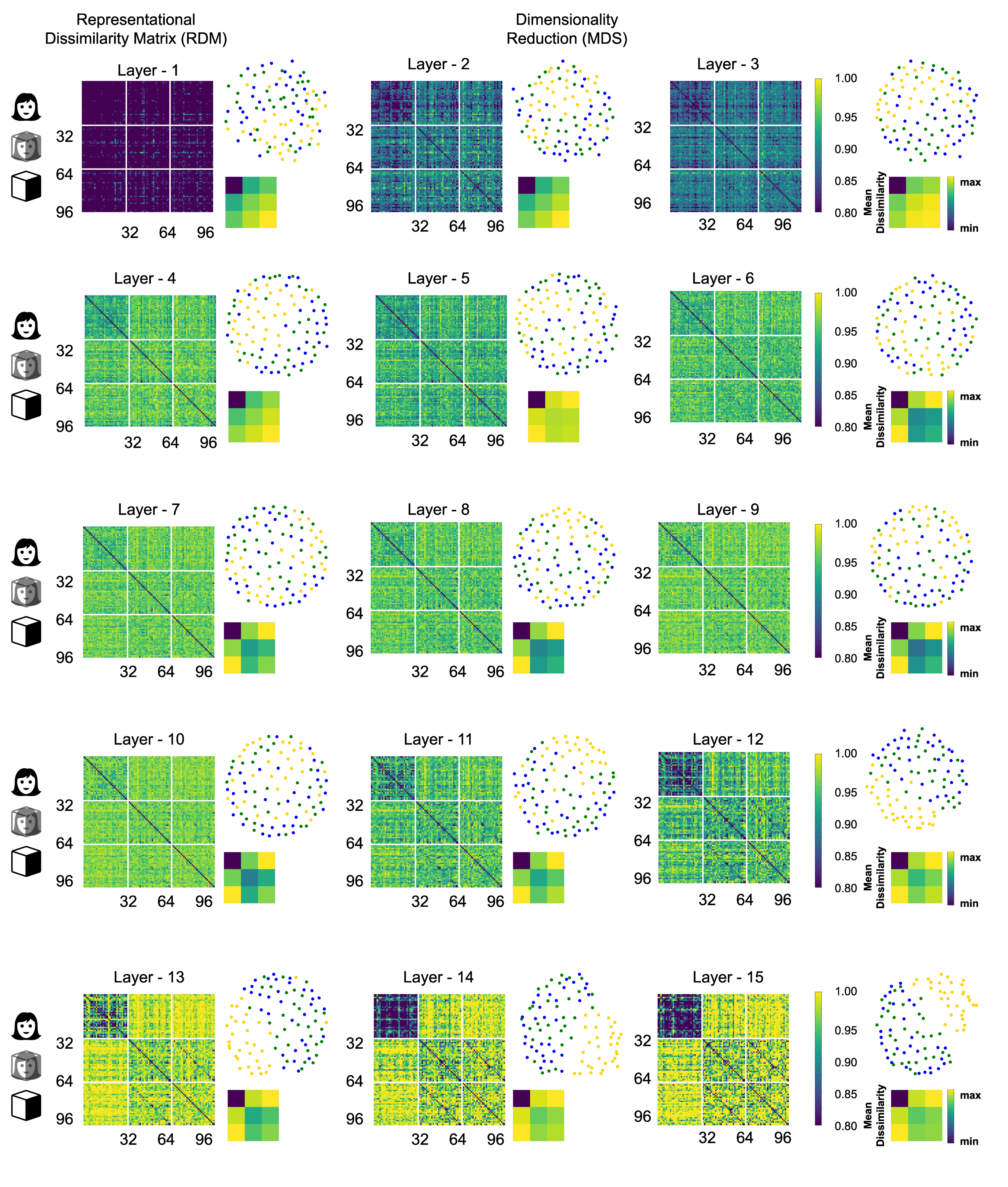

Supplement: S1 Fig — The Dual-task CNN’s representational dissimilarity matrices (RDMs) for the stimulus set of faces, pareidolia faces, and matched objects in all CNN layers are shown here. Dimensionality reduction of the RDMs using Multidimensional Scaling (MDS) is shown to the right of each RDM along with the category-specific RDMs constructed by averaging the corresponding larger matrix. The icons used in this figure have been obtained from The Noun Project (https://thenounproject.com/) under a royalty-free license. (TIF) [file pcbi.1012751.s001.tif]

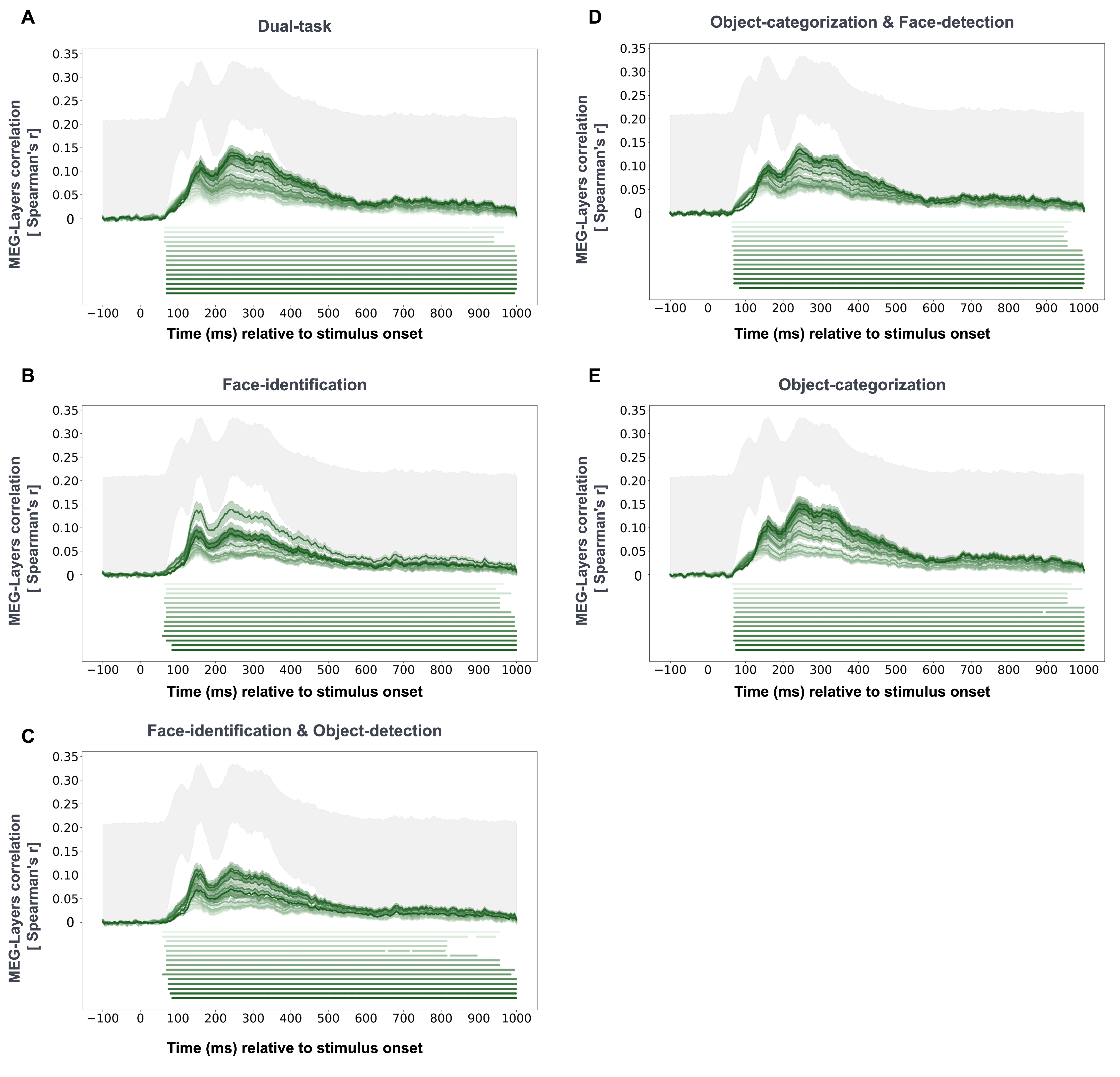

Supplement: S2 Fig — Each panel shows the correlation between MEG data and neural network layers over time relative to stimulus onset. Green lines represent individual layers, with darker shades indicating deeper layers (15-darkest). The gray shaded area represents the noise ceiling. Horizontal lines below each plot indicate periods of significant correlation for each CNN. (TIF) [file pcbi.1012751.s002.tif]

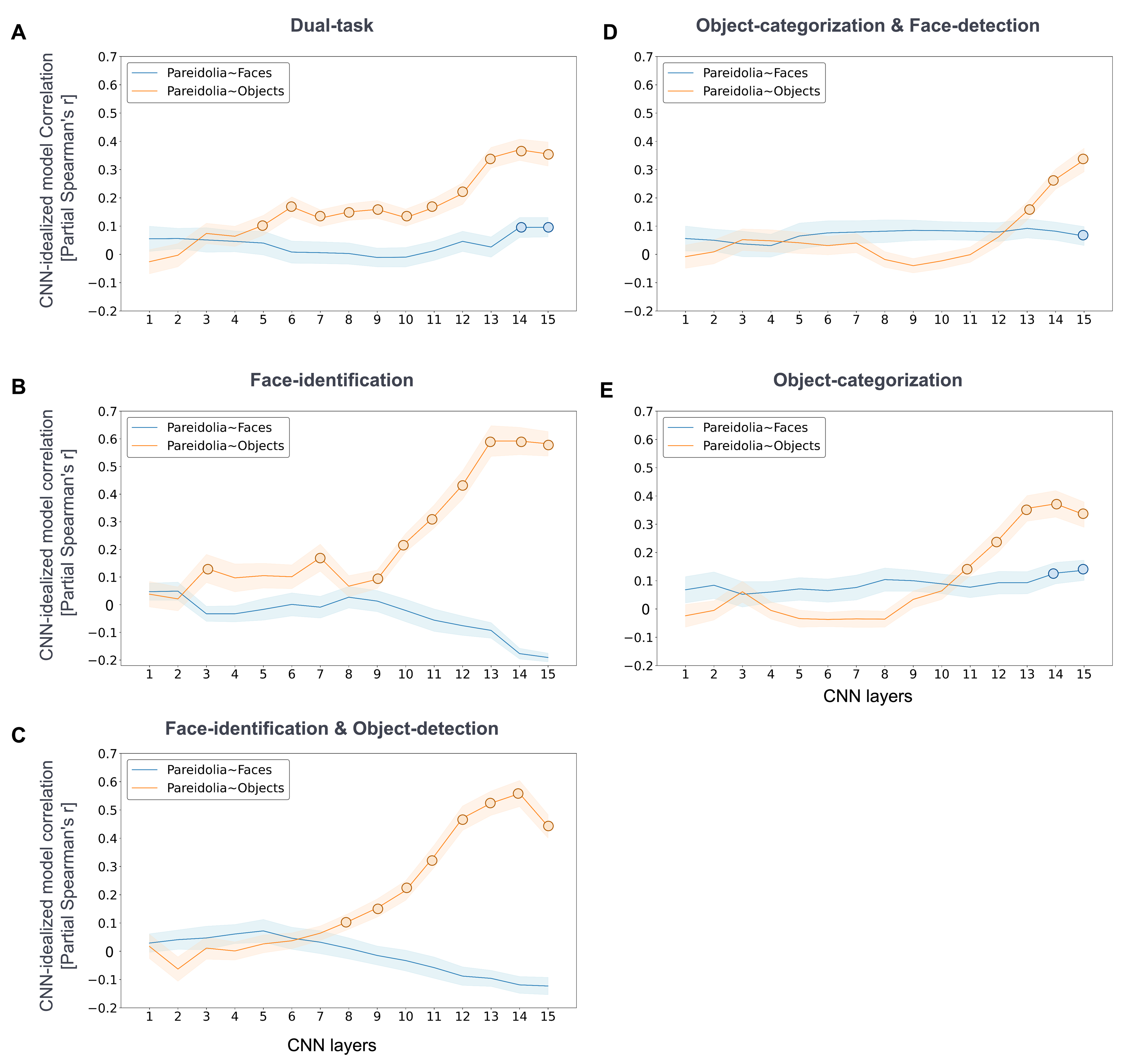

Supplement: S3 Fig — To assess the impact of low-level visual differences, such as luminance and contrast, among the stimuli, we conducted control analyses using the SHINE toolbox [73]. This step was crucial to ensure that any observed differences in representations were not merely due to these low-level features. In these analyses, we matched the low-level features of the face, object, and pareidolia stimuli across the different models: For the ‘Pareidolia ~ Objects’ model RDM (represented in orange), the representation remained robust even after adjusting for low-level features. Conversely, for the ‘Pareidolia ~ Faces’ model RDM (represented in blue), the representation dipped substantially after controlling for these features. These findings indicate that low-level features in non-controlled images play a crucial role in driving the perception of face pareidolia across all CNNs. This underscores the importance of controlling for such variables to isolate higher-level cognitive processes involved in face and object recognition. The shaded areas represent the standard error of the mean (SEM), bootstrapped across images. Colored circles indicate layers with significant correlations, determined using two-sided permutation tests with Bonferroni correction (p < 0.05). (TIF) [file pcbi.1012751.s003.tif]
